# What do cellular responses to acidity tell us about cancer?

**DOI:** 10.1007/s10555-021-10005-3

**Published:** 2021-11-30

**Authors:** Wiktoria Blaszczak, Pawel Swietach

**Affiliations:** Department of Physiology, Anatomy & Genetics, Parks Road, Oxford, OX1 3PT England

**Keywords:** Metabolism, pH, Acid–base, Cell lines, Variation, Adaptation, Selection, Evolution, Phenotype

## Abstract

The notion that invasive cancer is a product of somatic evolution is a well-established theory that can be modelled mathematically and demonstrated empirically from therapeutic responses. Somatic evolution is by no means deterministic, and ample opportunities exist to steer its trajectory towards cancer cell extinction. One such strategy is to alter the chemical microenvironment shared between host and cancer cells in a way that no longer favours the latter. Ever since the first description of the Warburg effect, acidosis has been recognised as a key chemical signature of the tumour microenvironment. Recent findings have suggested that responses to acidosis, arising through a process of selection and adaptation, give cancer cells a competitive advantage over the host. A surge of research efforts has attempted to understand the basis of this advantage and seek ways of exploiting it therapeutically. Here, we review key findings and place these in the context of a mathematical framework. Looking ahead, we highlight areas relating to cellular adaptation, selection, and heterogeneity that merit more research efforts in order to close in on the goal of exploiting tumour acidity in future therapies.

## The role of cell lines in cancer research

The World Health Organization estimates that cancer accounts for one in six deaths globally [1]. This headline figure is the reason for the tremendous and increasingly interdisciplinary research efforts aimed at understanding and treating the disease. The widespread laboratory use of cancer cell lines established from human patients has, beyond doubt, contributed greatly to the relative success of scientific research in improving survival for patients diagnosed with melanoma, Hodgkin lymphoma and breast, prostate, testicular, cervical, and thyroid cancers [2]. Despite emerging concerns and recognised limitations [3–6], cell lines offer a degree of insight into molecular disease mechanisms that is matched by no other available biological resource [7]. As research methods improve in terms of cost-effectiveness, spatiotemporal resolution, sensitivity, and data throughput, it is likely that cell lines will remain a cornerstone of cancer research in the foreseeable future, especially considering the growing consensus for repeating measurements on a wide panel of lines. In light of the so-called reproducibility crisis [8, 9] and the perceived failure of many preclinical concepts to succeed in human trials [10], the case for designing informative, relevant, and well-controlled experiments on cell lines has never been more pressing. This ambition can be met by considering clinical observations made on patients and by referring to predictions made by mathematical models which have their roots in ecology.

## What cancer patients and mathematical models tell us about the optimal design of experiments using cell lines

Cancer cell lines carry with them a record of genetic mutations which were ultimately responsible for the neoplastic growth that mandated surgical resection [11]. On excision, the cancer cells lost the unique cellular and humoral context that they grew into, which inadvertently impacts their biological behaviours *in vitro*. After many months or even years of passages in culture media, cell lines may behave very differently from the original cancer cells and even diverge between different laboratories [12–15]. These issues highlight the importance of two matters pertaining to good laboratory practice. Firstly, cell lines must be monitored for characteristic behaviours that verify the retention of key properties of the original cancer cells. Secondly, culture conditions should attempt to mimic the chemical (e.g. ionic composition [16–18]) and physical properties (e.g. matrix stiffness [19–21]) of the tumour [22, 23].

Historically, observations made on human cancers have provided essential instructions on how to perform *in vitro* experiments using cell lines. Over time, these observations have been refined and expanded to include newly described properties. This information includes a description of the hallmarks of cancer [24, 25], which experimental models should strike to retain in order to ensure disease relevance. Additionally, measurements on human tumours have described the physicochemical properties of the tumour microenvironment [26–29], such as the oxygen partial pressure (pO_2_) [30, 31] or acid–base balance quantified in terms of pH [32–35]. For example, elevated lactic acid production has been recognised – for over a century – to be characteristic of tumours, even when adequate oxygen supply would seem to favour oxidative phosphorylation [36]. This hallmark is exploited diagnostically in ^18^F fluorodeoxyglucose imaging to identify cancer cells based on their glucose appetite [37–39]. Thus, it is prudent to confirm that the cell lines chosen for studying a particular cancer also phenocopy its metabolic profile [40, 41]. It is equally important to ensure that culture conditions are relevant to the *in situ* milieu of the relevant tumour of interest. The reference thresholds for experimental conditions have been defined by measurements using invasive probes [42] and imaging of patient tumours [43] or by assays performed on biopsies [44, 45]. While these tasks are conceptually simple with contemporary technology, efforts to define precise thresholds are compounded by variation among patients (exacerbated further by any comorbidity), the spatiotemporal heterogeneity within tumours, and the diversity of metastatic niches. Consequently, it has not been possible to match every commonly used cell line with a bespoke set of growth conditions, although formal guidelines and informal agreements have been implemented in recognition of this issue [46–48].

A central pillar of the scientific process is to study how cells respond to controlled changes in specific factors. This hypothesis-driven, reductionist approach is necessary for ranking the various possible factors that influence cancer progression. However, this strategy raises the critical question of which factor to select as the independent variable for testing. It would be desirable to perform a sensitivity analysis for all known variables, but this is still outside the scope of modern research approaches. Instead, decisions on what factor to vary have often been influenced by historical precedent, methodological constraints, or even personal biases. One approach that can assist in identifying the dominant factors influencing carcinogenesis involves mathematical models that have their origins in studies of interactions between species in an ecosystem [49–52]. Despite some obvious differences between animal and cellular societies, the results of these simulations have laid down priorities for cancer research.

**Fig. 1:**
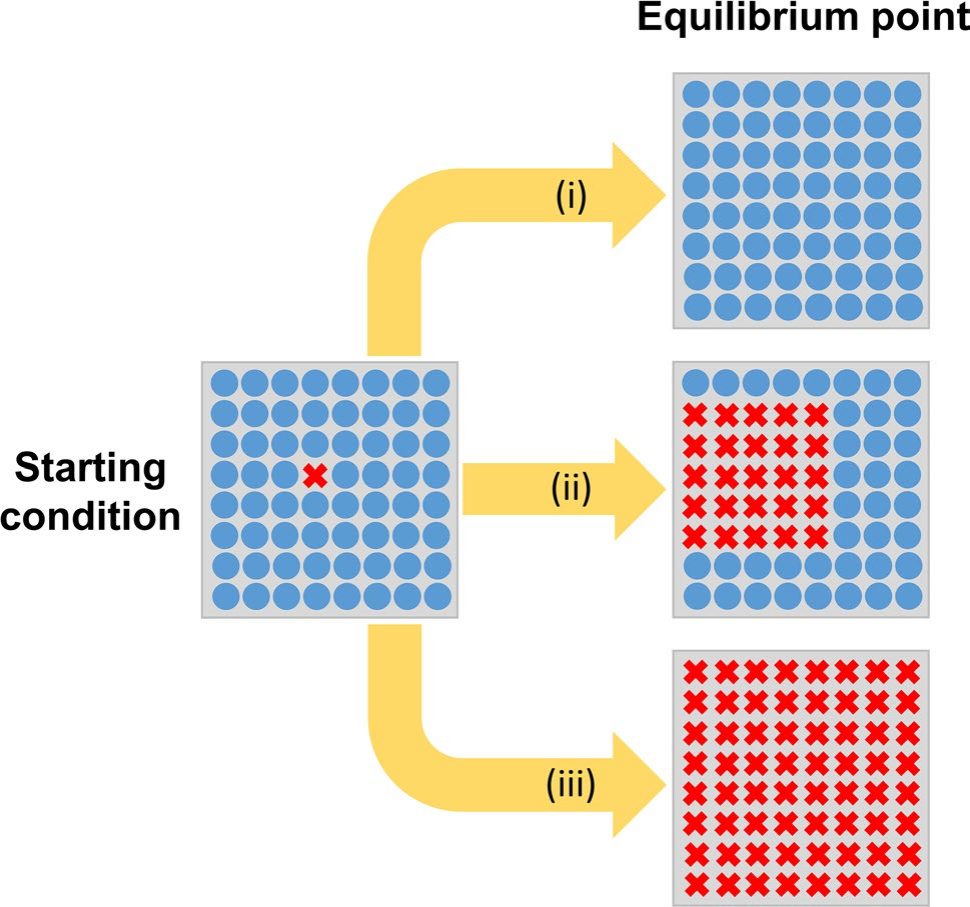
Predicted outcomes of Lotka–Volterra models. Cancer cells (red-crossed) and normal cells (blue circles) cohabit a tissue ecosystem. With time, the interaction between these cells and their environment can lead to three trajectories that culminate in three different equilibrium points: (i) extinction of cancer, i.e. cancer remission, (ii) stable coexistence, i.e. benign tumour, (iii) cancer cells take over normal cells, i.e. invasive cancer

The relationship between cancer cells and normal cells resembles predator–prey interactions, described mathematically using a system of differential equations called the Lotka–Volterra equations [53]. The elegance of these simulations is that they can track the tumour-host interplay over time until one of three scenarios is attained at the equilibrium point (Fig. 1):

(i) extinction of cancer cells, i.e. cancer recedes;
(ii) a stable coexistence of cancer and normal cells, i.e. a benign tumour;
(iii) unconstrained growth of cancer cells leading to the extinction of normal cells, i.e. invasive cancer.

**Fig. 2:**
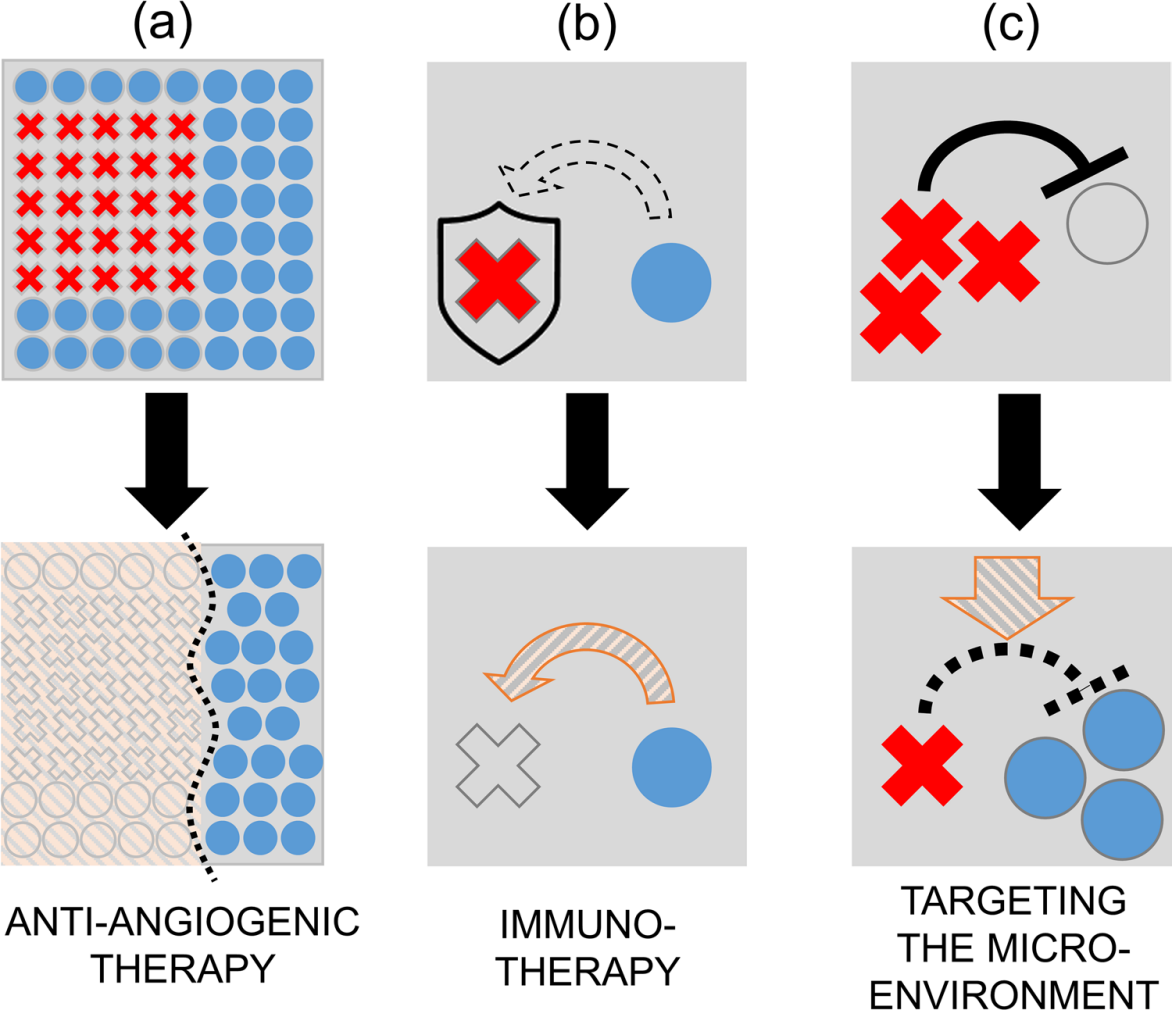
Effective strategies for cancer therapy predicted by mathematical models. Cancer cells (red-crossed) and normal cells (blue circles) cohabit a tissue ecosystem. **A** Reducing the capacity of the tissue to carry cancer cells, e.g. with antiangiogenic therapy. **B** Weakening cancer defence mechanisms against host activities, e.g. immunotherapy. **C** Preventing cancer cells from generating a harsh environment for the host cells, e.g. influencing the microenvironment

In terms of therapy, it is important to know which trajectory a particular carcinoma will follow because of tangible opportunities for drugs to reroute cellular fates towards the first or at least second outcome. These mathematical models have a distinct advantage over clinical observations and cell line-based experiments in that they are able to ‘explore’ the process of carcinogenesis from an early stage, provided that there is sufficient data at other time points to best-fit values to all necessary variables. Insight into this longitudinal process is not normally available by studying patients because many cancers (particularly pancreatic ductal adenocarcinoma [54]) are detectable only as they approach the equilibrium state. In the laboratory, it is not normally possible to follow a system over a sufficiently long time for it to truly attain a steady state because of inherent limitations in the design of *in vitro* and animal experiments. Moreover, the cell lines established from patients capture the latter part of the carcinogenesis process, giving limited insight into how the cells got to that particular point. In the absence of empirical evidence, mathematical models have been consulted to give a steer on the best strategies to interfere with the early stages of cancer growth. Examples of interventions predicted by mathematical modelling to be effective include those that target one or more of the following (Fig. 2):

- growth-related interventions that restrict the host’s capacity to harbour cancer cells;
- host defences against cancer cells, such as the immune response;
- the negative effect that cancer cells exert on host cells, e.g. cancer cell activities that create a harsh microenvironment for the host tissue.

The clinical effectiveness of antiangiogenic therapies and immunotherapy can be explained in terms of their actions on the first and second targets, respectively [55, 56]. The third target is recognised for its untapped therapeutic potential that could be realised by studying the interplay between cell lines and their environment. Frustratingly, the nature of this interplay is still inadequately characterised and, understandably, has not yet achieved the same level of success in clinical trials as antiangiogenics or immunotherapy. Since mathematical models predict synergy between two or more of the above targets, all three targets merit balanced research efforts.

Mathematical models are also useful for predicting the long-term effectiveness of therapies formulated on the basis of promising preclinical results. These simulations have often arrived at counterintuitive conclusions. For example, according to Lotka–Volterra models, therapies that selectively kill the majority of cancer cells cannot, in the end, change overall outcomes. This is because the ‘ecosystem’ eventually arrives at the same equilibrium point once the surviving population of cancer cells expands [57–59]. In other words, even a small number of surviving cells is able to repopulate tissues and continue along the prior trajectory.

## The case for studying acidosis in cancer research

Mathematical predictions highlighting the therapeutic potential of targeting the cell-(micro)environment interplay have reinvigorated efforts to understand how cancer cells influence their surrounding milieu and how this feeds back on cancer and normal cells growing alongside one another. Historically, the two aspects of the harsh tumour microenvironment that have garnered considerable scientific attention are hypoxia and acidosis, ostensibly because of their direct relationship with mitochondrial respiration and glycolytic metabolism, respectively. Cancers cannot grow without an adequately high metabolic rate, thus some combination of lactic acidosis and hypoxia is expected in solid tumours [36, 60–62]. Of these two chemical variables, it is more conceivable to exercise experimental or therapeutic control over the extracellular pH of tumours (pHe), as compared to pO_2_; for example, by means of pH buffering [63] described later. Another argument in favour of targeting pHe is that the remit of such interventions is largely restricted to the extracellular compartment. This selectivity has the beneficial effect of protecting intracellular pH (pHi) in noncancerous cells, although some degree of pHi-pHe coupling is inevitable [64]. In contrast, O_2_ gas permeates freely across membranes; thus, any manipulation to extracellular pO_2_ will seamlessly transmit across to the intracellular compartment of both cancerous and noncancerous cells [65].

**Fig. 3:**
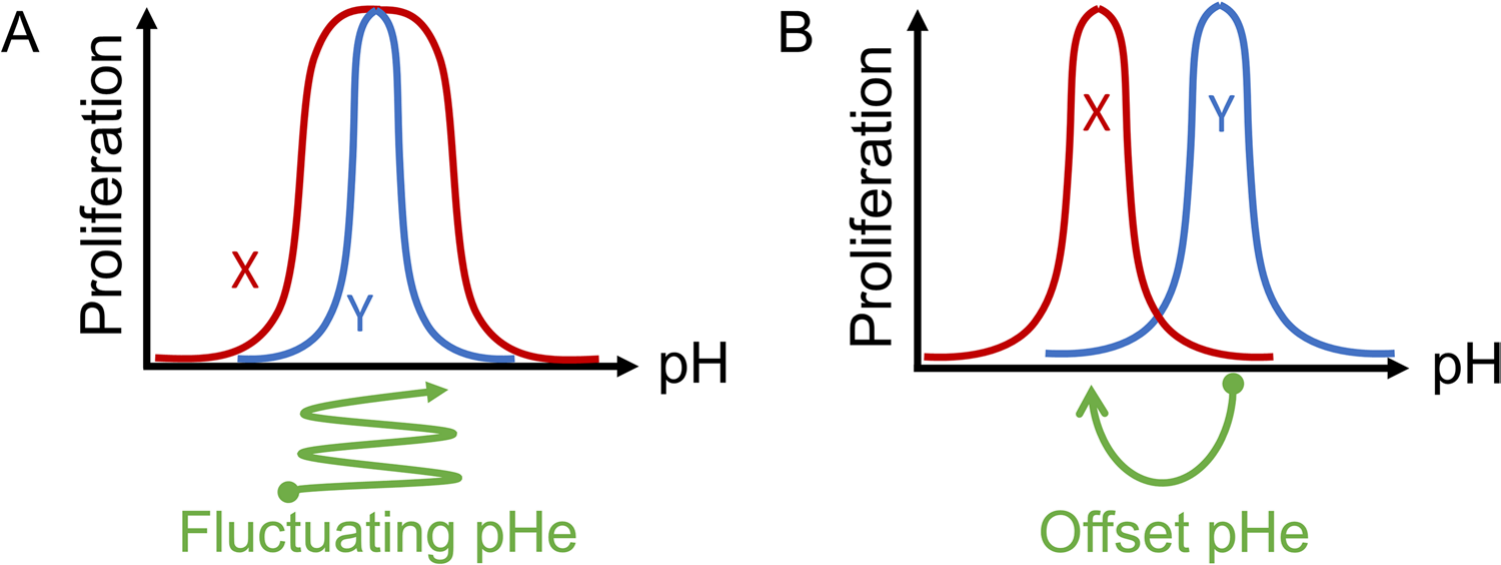
Examples of pH sensitivity curve for proliferation. **A** Cell *X* has a broader pH optimum compared to cell *Y*, therefore its survival prospects are higher during fluctuations in pH. Such dynamic changes in pH have been described in tumours. **B** Cell *X* has an acid-shifted pH optimum compared to cell *Y*. If cell *X* also has a higher metabolic rate, it is likely to drive tissue pHe to a lower level. This would have the effect of giving cell *X* a survival advantage over cell *Y*

Some of the earliest experiments investigating the effect of pHe on cancer cells were performed by Harry Eagle in the 1970s [66]. His results, using readouts such as proliferation, introduced the concept of a pHe optimum range. A key observation borne from this and many subsequent studies was the steepness of pHe survival curves for many cancer cell lines [67]. The reason underpinning this exquisite sensitivity relates to the multitude of protein targets for H^+^ ions. The significance of this observation is that only a small change in pHe is sufficient to produce a meaningful effect on cell behaviours. Thus, among competing cell populations, the shape of their pHe sensitivity curves can have a profound impact on survival prospects. For example, a cell with a broader pHe optimum range would be more successful during fluctuating pHe, as compared to a cell with a narrow pHe optimum range (Fig. 3A). There are also various adaptive strategies based on pHe sensitivity that could give a competitive advantage to a particular subpopulation of cells. For instance, cancer cells could gain a selective advantage over host cells if their pHe optimum range is shifted towards a more acidic level and, concurrently, their glycolytic output is increased. This combination is expected to reduce pHe to a level that is harsh for host cells but conducive for the adapted cancer cells (Fig. 3B). This strategy relies on the ability of cancer cell populations to change beyond the normal constraints imposed on noncancerous cells. Critically, only minor adjustments to pHe sensitivity may be sufficient to change the trajectory of cancer towards invasive behaviour. By the symmetrical argument, only a small degree of alkalinisation, such as that attainable with therapeutic buffering [68], may be required to steer away from the malignant phenotype.

Changes in pHe can produce a myriad of consequences on cell biology [69–72] because protonation is a form of posttranslational modification that affects virtually all proteins [73]. Notwithstanding the breadth of potential targets, a subset of proteins has been described as bona fide pHe sensors, whose primary role is to transduce a pHe signal into a measurable cell response [72]. The discovery process for these sensors has typically taken the approach of mapping the pHe sensitivity of downstream readouts. For example, the first H^+^-sensing G-protein-coupled receptor OGR1 was discovered by screening for effects on the second messenger IP_3_ [74]. These, and many other studies, were biassed by design, in the sense that the hypothesis was testing the pHe sensitivity of an *a priori* defined target, rather than taking a more agnostic approach towards the identity of the putative H^+^ sensor. To address this concern, recent studies have sought evidence for pHe-sensitive pathways using unsupervised discovery pipelines, such as transcriptomics [75, 76], proteomics [76, 77], or metabolomics [78, 79]. The general conclusion from these studies is that a decrease in pHe triggers a multifaceted response that involves a change in phospholipid composition [79], increases glutaminolysis and fatty acid synthesis [78], induces autophagy [80, 81], remodels the extracellular matrix [75, 82], modulates the cell cycle [75], affects DNA repair [75], promotes epithelial-mesenchymal transition (EMT) [83], and increases alternative splicing [84]. However, when interpreting these results, it is important to consider the precise level of pHe attained during measurements and how this change was made experimentally. Since pH is a continuous variable, the measured response must refer to the specific ‘test level’. This becomes problematic if pHe is not noted when data are collected or when pHe is poorly controlled, e.g. is drifting because of ongoing metabolism or if ambient CO_2_ levels are changed between incubators and bench side assays. There are multiple options for changing the pHe of media, and it is important to relate these to the physiological scenario. Normally, a fall in pHe arises from an increase in CO_2_ partial pressure (so-called respiratory acidosis) or a decrease in [HCO_3_^−^] due to a buildup of nonvolatile (i.e. non-CO_2_) acids or renal loss of HCO_3_^−^ ions (so-called metabolic acidosis) [85]. Metabolic acidosis must be accompanied by an increase in the concentration of the anion that stoichiometrically accompanies H^+^ ions (the so-called conjugate base), for example, lactate anions. This organic component may, on its own right, produce cellular responses; indeed, some metabolic responses appear to depend on whether they are evoked by lactic acidosis or acidification paired with a different anion [78]. Thus, the interpretation of acid responses becomes difficult if the study write-up did not explain how pHe was reduced. Substantial differences in the experimental process for changing pHe can make comparisons between studies difficult or even impossible, especially when apparently contradictory findings are postulated [86, 87]. To avoid these issues, adherence to recently published guidelines is recommended [46].

A large number of studies have documented the actions of acidity on cancer cells. However, many of these reports have not consistently described the method by which pH had been manipulated or provided a justification for a particular choice of pHe [70, 71, 88–91]. This is problematic because different strategies for manipulating pH can produce distinct responses, even if the final pHe is matching. Table 1 presents a selection of pH-related studies alongside information on how pHe had been changed. The most common way of creating acidic conditions is to exchange NaHCO_3_-based media with formulations containing nonvolatile buffers, as HEPES and PIPES, followed by titration with strong acids or bases. This can be problematic because media titrated outside CO_2_ incubators will inevitably acidify in an atmosphere of 5% CO_2_, thus compromising pH control. In a smaller subset of studies, pHe was adjusted by changing the concentration of NaHCO_3_, a strategy that we argue is most physiological, predictable, and stable. In half of the primary literature reviewed herein, the technique used for adjusting pH was not reported with adequate detail. The lack of consistent reporting standards continues to be problematic for establishing a unifying model of how acidosis affects cancer cells.

**Table 1:** Examples of acidity-triggered cellular responses

*Model*	*Method of manipulating pHe*	*Duration under acidosis*	*Target pHe*	*Reported cell response*	*Reference*
Breast cancer: MCF‐7, MCF10‐AT, MDA‐mb‐231, MCF10, and MCF10AT	Medium supplemented with 25 mmol/L of PIPES and HEPES and the pH adjusted to 7.4 or 6.7	Acute: 72 h and prolonged: 3 months	6.7	Resistance to anoikis, elevated collagen production, upregulated ECM remodelling enzymes: TGM2, LOXL2	[82]
Colorectal cancer: HCT116, SW480, LoVo, SW620, and HT29	Medium maintained in a 5% CO_2_ atmosphere at 37 °C. The medium was supplemented with 25 mM HEPES and PIPES and the pH was adjusted	Acute, 24 h	6.5	Increased ASIC2 expression, ASIC2-driven invasion	[92]
Glioma: U87MG, T98G, and U251	Medium supplemented with 25 mM HEPES and the pH adjusted to 6.8; 6.7; 6.6; and 6.5	Acute, 24 h	6.8, 6.7, 6.6, 6.5	Induced stem-cell phenotype, increased OXPHOS, upregulated IL22, GUCA2B, CYP24A1, OR6P1	[93]
Breast cancer: MCF-7, ZR-75–1, T47D, MDA-MB-231, and MDA-MB-157	Medium adjusted to acidic pH with HCl	Acute, 24 h	6.7	Metabolic reprogramming to oxidative PPP and glutaminolysis	[94]
Pancreatic cancer: PANC-1, AsPC-1; cervical cancer: HeLa**;** glioma: T98G; colorectal cancer: SW620	pH was manipulated by varying concentrations of NaHCO_3_ in CO_2_-rich atmosphere. pH 7.4: 8 mM, pH 6.8 2 mM	Acute, 24 h	6.8	Decreased adhesion, SREBP2 activation, IDI1 and PDK4 upregulation	[86]
Mesenchymal stem cells; melanoma: A375M6	Medium adjusted to acidic pH	Acute, 24 h	6.6–6.7	Low pH-exposed MSC enhanced *in vivo *melanoma growth, acidosis of tumour microenvironment potentiated the pro-tumoral activity of MSC	[95]
Melanoma: MV3	NaHCO_3_-free medium supplemented with HEPES (10 mmol/L) and adjusted to the respective experimental pH	Acute, 24 h	7.0; 6.8; 6.4	Reduced cell to cell adhesion, invasion, increased cell-surface adhesion	[87]
Breast cancer: MCF7, SUM52PE	Medium supplemented with 25 mM HEPES, adjusted to the acidic pH	Acute, 24 h	6.7	Inhibition of canonical hypoxia response and activation of UPR and inflammation	[96]
Glioblastoma: U87-MG, HTB-14; cervical cancer: HeLa, CCL-2; Mouse Lewis lung carcinoma: LLC1, CRL-1642; glioma: GL261	Medium adjusted to acidic pH	Acute, 24 h	6.6; 6.2; 6.0	Increased uptake of lipoproteins via proteoglycan-dependent endocytosis	[97]
Glioblastoma: LN229	Medium adjusted to desired pH with 2 N HCl	Acute, 3 h	6.2; 3.4	Increased surface cholesterol elevated proliferative and stem-like potential	[98]
Breast cancer: MCF-7, MDA-MB-468, MDA-MB-231, and SkBr3	Medium adjusted to pH 6.5	Acute, 48 h	6.5	Decreased glycolysis, elevated glutaminolysis and fatty acid synthesis	[78]
Melanoma: Me30966, Mel501, WM793, A375, SK-Mel-28	pH was manipulated by varying concentrations of NaHCO_3_ in CO_2_-rich atmosphere	Acute, up to 24 h	6.8, 6.5	Induced autophagy, mTOR inhibition, activated AMPK, reduced glucose and amino-acid uptake	[80]
Breast cancer: MDA-MB-231, MCF7; pancreatic cancer: PANC-1	pH was manipulated by varying concentrations of NaHCO_3_ in CO_2_-rich atmosphere; the osmolality was maintained by adjusting NaCl	Prolonged, 1 month	6.5	Metabolic deregulation, ECM remodelling, altered cell cycle regulation, induced DNA damage response. Elevated expression of SCNN1A, CACNG4, ASIC1, SCN1B, IFITM1	[75]
Melanoma: C8161	Slow conditioning over the course of 1.5 months with 0.15 pH units drop every 2 weeks	Prolonged, 1.5 months	6.7	Increased invasion, motility, altered gene expression relating to: cell cycle, inflammation, Wnt signalling, apoptosis, IL-2, cell adhesion	[99]
Lewis lung carcinoma: LLCm1	Cells were adapted to acidic pH by serial passaging through media of stepwise decreasing pH (7.0, 6.8, and 6.5) until pH 6.2 was reached. The cells were maintained for 2–4 weeks at each pH	Prolonged, 3 months	6.2	Increased metastatic activity through MMPs, increased migration and invasion	[100]
Colorectal cancer: SW480, SW620	Medium supplemented with 25 mM HEPES and PIPES, pH adjusted to 6.5	Prolonged, 3 months	6.5	Increased invasion and metastasis, altered chromatin accessibility including DNA remodelling-associated pathways, HDAC, SIRT1 pathway, DNA methylation	[101]
Breast cancer: MDA-MB-231, HS766T	Cells were cultured and passaged directly in acidic medium	Prolonged, 3 months	6.7	Cytoplasmic vacuolated phenotype, elevated autophagy, in mouse model autophagy was reduced by systemic treatment with sodium bicarbonate	[81]
Melanoma: Mel501; breast cancer: MCF7ac; prostate cancer: PC3ac	Cells allowed to acidify in unbuffered medium for 5 days, then passaged and moved to a medium of pH 6.5	Prolonged, 3–4 weeks	7; 6.75; 6.5	Remodelled lipid composition towards longer, unsaturated acyl chains, upregulation of genes involved in acyl chain desaturation, elongation and phospholipid transfer	[79]
Melanoma: Me30966; prostate cancer: LNCaP; osteosarcoma: SaOS2; breast cancer: SKBR3; colorectal cancer: HCT116	Cells allowed to acidify in unbuffered medium for 5 days, then passaged and moved to medium of pH 6.5	Prolonged, 3–4 weeks	6.5	Increased exosome release	[102]
Cervical cancer: SiHa; head and neck cancer: FaDu; colorectal cancer: HCT-116	Medium supplemented with 25 mmol/L of both PIPES and HEPES, pH adjusted to 6.5	Prolonged, 8–10 weeks	6.5	Increased mitochondrial protein acetylation, switch to fatty acid oxidation	[103]

Historically, tumours have been considered acidotic and hypoxic, but this does not imply that regions of low pO_2_ also have low pHe. Indeed, recent evidence argues for distinct areas of acidosis or hypoxia, each associated with distinct cellular behaviours *in situ* [84]. At the cellular level, responses to hypoxia tend to be different to those evoked by acidosis, which is consistent with the unique molecular transducers of low pHe or low pO_2_ responses [104, 105]. Very few studies have offered insight on how responses to pHe are subservient to pO_2_ [97], and vice versa [94, 106]. This interaction is highly relevant to our understanding of cancer responses at the interface between two selection pressures, a niche that may favour the most invasive of phenotypes.

## Acid-driven selection versus adaptation

**Fig. 4:**
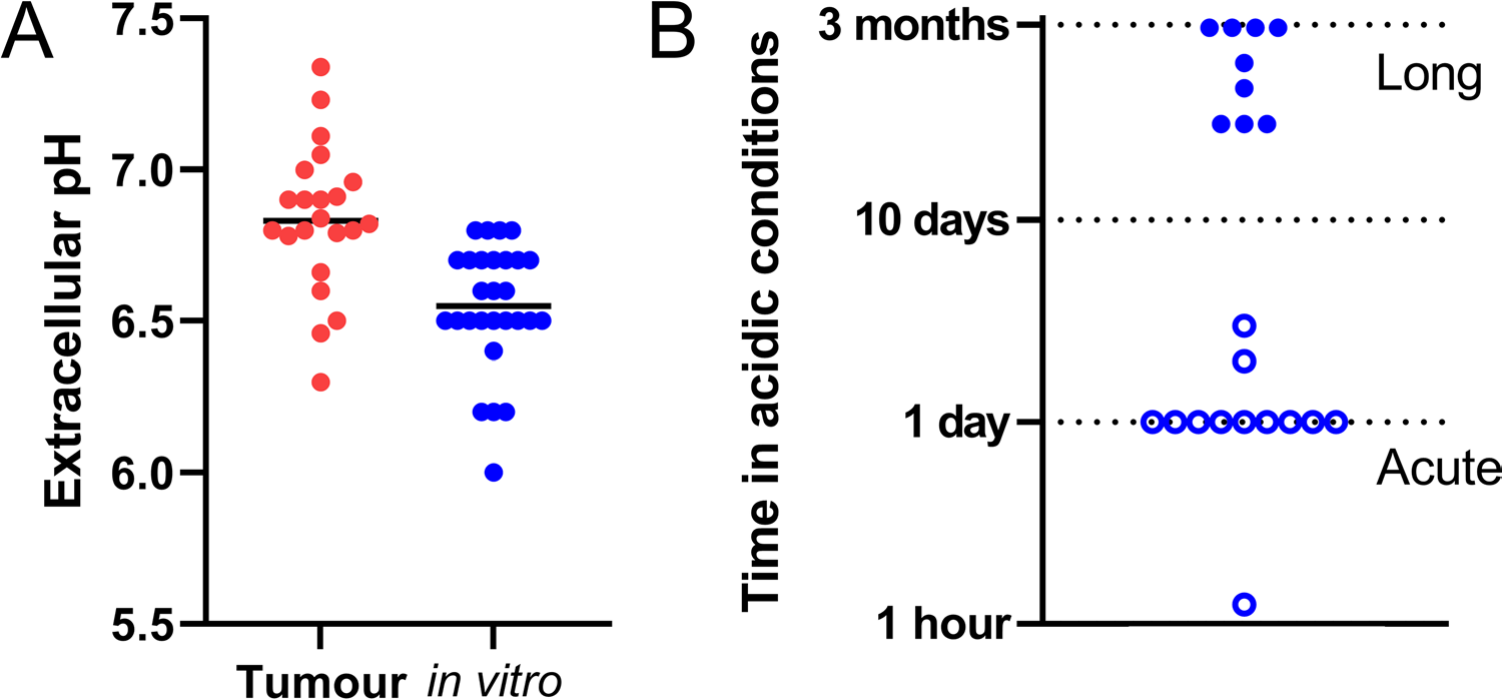
Analysis of literature related to acidity and cancer. **A** Comparison between the extracellular pH reported within solid tumours (red) and pHe values selected for *in vitro* studies (blue). **B** Time under acidic conditions in studies using cultured cancer cell lines. The primary literature referred to these as long or short exposure, as indicated by empty and filled circles, respectively. References used in these analyses are listed in Tables 1 and 2

While the pHe sensitivity of cancer cell biology is undisputed, a more controversial issue concerns the level of pHe that should be considered ‘typical’ for tumours. Unlike most normal tissues that maintain their interstitial pHe near 7.4 by means of good capillary perfusion, there is no single value of pHe that could be considered representative of tumours. MR imaging modalities, ranging from 3-aminopropylphosphonate spectra [107] to CEST [108] have reported tumour pHe to be as low as 6.3 [109], although the spatial resolution of these techniques may inadvertently smoothen steep gradients of pHe and therefore mask small pockets of profound acidity. A compendium of pHe measurements using imaging and electrodes is provided in Table 2. This meta-analysis shows that most intratumoural pHe measurements have been in the range 6.4–7.4, with a median value of 6.8 [110–113]. Accordingly, experiments using cell lines under culture conditions should avoid exceeding these limits. Despite awareness of this concern, there has been a tendency for *in vitro* studies to undershoot the pHe range in tumours (Fig. 4A). To understand what level of pHe is appropriate for a cancer cell line, it is first important to recognise the factors responsible for setting the level of acidosis in a tumour and the degree to which this could be manipulated therapeutically.

**Table 2:** Extracellular pH reported in tumours. Measurement methods are chemical exchange saturation transfer (CEST), magnetic resonance imaging (MRI), biosensor imaging of redundant deviation in shifts (BIRDS), paramagnetic chemical exchange saturation transfer (PARACEST), ^13^C-labelled zymonic acid (ZA), variable radio frequency proton-electron double-resonance imaging (VRF PEDRI), electron paramagnetic resonance (EPR), and pH microelectrode

*Model*	*Method*	*pHe*	*Reference*
Breast cancer (MMTV-Erbb2 transgenic mice)	CEST-MRI	6.30–6.90	[109]
Hepatoma (McA-RH7777)	CEST-MRI	~ 6.80	[109]
Glioblastoma	BIRDS	6.90 ± 0.01	[110]
Bronchial tumours	pH-microelectrode	6.46 ± 0.35	[111]
Nonmetastatic breast cancer (TUBO)	CEST-MRI	6.84 ± 0.03	[114]
Triple negative breast cancer (4T1)	CEST-MRI	6.79 ± 0.02	[114]
Metastatic breast cancer (TS/A)	CEST-MRI	6.80 ± 0.03	[114]
Spontaneous lobular carcinoma (BALB-neuT)	CEST-MRI	6.96 ± 0.03	[114]
Hepatic carcinoma	CEST-MRI	6.66 ± 0.19	[115]
Hepatic hemangioma	CEST-MRI	7.34 ± 0.09	[115]
Glioma (U87)	CEST-MRI	7.00 ± 0.1	[116]
Glioma (U87)	CEST-MRI	6.60 ± 0.1	[116]
Breast cancer (MCF-7)	PARACEST-MRI	~ 6.50	[117]
MATB III adenocarcinoma	ZA-MRI	6.82–7.11	[118]
Breast cancer (C57Bl/6 Met-1)	VRF PEDRI	6.80 ± 0.10	[119]
Pancreatic cancer (MIA-PaCa-2)	EPR	~ 7.05	[120]
Pancreatic cancer (SU.86.86)	EPR	~ 6.90	[120]
Pancreatic cancer (Hs766t)	EPR	~ 6.91	[120]
Prostate cancer (LNCap)	CEST-MRI	6.78 ± 0.29	[121]
Prostate cancer (PC-3)	CEST-MRI	7.23 ± 0.10	[121]

pHe is ultimately set by the rate of metabolic acid production, working against the capacity to clear this with blood perfusion. These two processes are coupled together by the diffusion of H^+^ ions across the extracellular compartment. In well-perfused tissues – where cell capillary distances are typically no more than a few microns – diffusive coupling is excellent. In this scenario, perfusion is expected to keep pHe close to 7.4, even if the metabolic rate is very high (e.g. as is the case in the myocardium). The situation can be radically different in tumours that have abnormal vascular penetration. Here, diffusion distances can expand to hundreds of microns, generating pHe gradients across the extracellular space. The molecular pathways responsible for these expanded diffusional distances relate to the extent of vascular penetration and are typically guided by hypoxia and evoked by factors such as vascular endothelial growth factor (VEGF) [122–127]. These processes become deranged in tumours, leading to inadequate and often fluctuating perfusion [128–130] and can be manipulated with drugs such as anti-VEGF therapies [131–134], which may exert at least a part of their action via a change in pHe. In such diffusion-limited environments, pHe can be driven downwards by metabolism, particularly by glycolysis because lactic acid is a stronger acid of slower mobility compared to mitochondrially generated CO_2_. The balance between these fluxes can be altered by inhibitors. For example, blockers of oxidative phosphorylation force cells to rely more on glycolysis [135]. Conversely, inhibitors of glucose uptake can lessen glycolytic rate and hence lactic acid production [136], as would inhibitor of monocarboxylate transporters (MCT), the main route of lactic acid efflux from cells [137, 138]. The effective diffusion coefficients of these acidic products will depend on the equilibration state of CO_2_/HCO_3_^−^, as this depends on the activity of exofacial carbonic anhydrases (CAs), such as CAIX or CAXII [139–145]. As described previously [146], the effect of CA activity on pHe depends on the nature of the disturbance. Net release of CO_2_ drives equilibrium towards hydration and thus CA activity would support further acidification [139]; in contrast, the release of H^+^ ions, such as in the form of lactic acid via monocarboxylate transporters (MCTs) would drive the reverse reaction, and CA catalysis would tend to raise pHe [146].

Metabolism will drive pHe to a lower level until it stabilises at a steady state. This scenario is reached when metabolic flux generated by cancer cells comes into balance with the diffusive flux across the poorly perfused tumour interstitium. As pHe falls, the diffusive gradient becomes steeper, which drives a larger diffusion flux of H^+^ ions. Eventually, this diffusive flux will match metabolic output and result in a steady-state pHe. The steady-state pHe could be attained at a less acidic level if metabolic rate became curtailed at low pHe; indeed, this is the case for glycolysis which is inhibited under acidic conditions [147]. Since glycolysis essentially ceases below pHe 6, tumour pHe is unlikely to fall below this level, as there is no obvious alternative source of acid that would drive pHe any lower. To summarise, tumour pHe depends on the rate of perfusion, diffusion distance, metabolic rate and its pH-dependence. It is important to consider these variables in the context of a particular tumour before designing experiments on cell lines that probe responses to acidosis.

Once an appropriate target pHe is determined for a particular investigation, the next step is to consider how to attain this acid–base balance experimentally. Various strategies for reducing pHe have been used in previous studies, ranging from an abrupt drop to the target pHe [98], to a more graded change over an extended period of time [82] (Fig. 4B). An alternative approach is to allow cancer metabolism itself to gradually acidify the milieu until the target pHe is attained [79, 102]. Although slow to reach its target, this method has the advantage that the source of acidity is endogenous and the final pHe is within the scope of the cell’s capacity to acidify its milieu. These three distinct pHe manoeuvres may produce radically different responses, even if the same end point pHe is reached because various degrees of acid selection and acid adaptation may take place. The interpretation of experimental outcomes must carefully consider the nature of the acid–base intervention.

A treatment will result in selection if it is survived by a subpopulation of cells only. This scenario would be favoured by large and abrupt pHe changes [148], such as those arising from a single medium change [98]. Although acid selection has been discussed at length in theoretical models of cancer [149–156], it is poorly characterised experimentally. Part of the reason is that the surviving cellular subpopulations may be very sparse and therefore inadequate for certain types of measurements. In contrast, adaptation will take place when the pHe change is slower because it gives cells time to respond. A dominance of acid adaptation over acid selection is most likely when metabolism is responsible for acidification because negative feedback circuits acting on metabolic rate protect against unmanageably fast pHe changes. The interpretation of experimental results must carefully consider the time line over which pHe was changed and include this information in the methodological write-up. Many of the experimental descriptions of acid responses may, in fact, relate to a combination of early-onset acid selection, followed by acid adaptation in the surviving cells. Some insight into these processes can be obtained by running parallel experiments that differ in the rate of pHe change, relative to cell doubling time. This is a worthwhile exercise because the distinction between acid selection and acid adaptation has implications on the phenotypic landscape of a tumour, and hence susceptibility to therapies. In principle, it would be feasible to vary tumour pHe dynamically or even impose bespoke waveforms that alter the balance between selection and adaptation and influence the evolution of cancer populations.

## Exploiting acidosis in cancer therapies

It is conceivable that some of the pro-oncogenic consequences of low pHe could be reversed by raising the pHe of solid tumours *in vivo*. This strategy is among the three predicted to have anticancer efficacy by evolution models (Fig. 2). The most thoroughly studied approach to increasing the pH within the tumour microenvironment is the systemic administration of bicarbonate. This so-called buffer therapy decreased metastasis formation [157], restored immune function [158], reduced tumour growth [159], and extended overall survival [160]. However, the issue with oral supplementation of bicarbonate in humans is the required dose, which is too high for comfortable administration thus severely limiting patient adherence [161]. To circumvent this compliance issue, a recent study replaced systemic administration with local delivery [162]; this significantly enhanced the efficacy of therapeutic transarterial chemoembolization, yielding 100% response in the combination-treated group. Some studies tested alternative buffering formulations, including Tris [163] and IEPA [164], but these are yet to be tested in a clinical setting. Another proposed way of raising pHe is the injection of urease, an enzyme that produces NH_4_^+^ and HCO_3_^−^ from urea, and thereby increases buffering locally. This intervention reduced tumour load and elevated the efficiency of chemotherapeutics in a mouse model of breast cancer [165].

An attractive therapeutic strategy that exploits pH combines the effect of pH changes on cancer cells and on immune surveillance. This approach targets two methods predicted to curtail cancer growth (Fig. 2). The effects of acidosis on immune function have been summarised by Damagaci et al. [166]. Briefly, extracellular acidity is deemed an immunosuppressor as it is associated with T-cell anergy [167]. The function of tumour-specific CD8^+^ T lymphocytes is impaired under acidic conditions, which manifests in decreased cytokine secretion and cytolytic activity [168]. Intriguingly, acidic niches in lymph nodes have a physiological role in suppressing resident T cells [169], and this mechanism is believed to be hijacked by solid tumours. Recent results confirmed the negative effects of acidosis on dendritic cells in terms of reduced migration, membrane, and cytoskeletal properties [170]. Similarly, high extracellular lactate concentrations, as an inseparable component to lactic acidosis, reduce migration and cytotoxicity of CD4^+^ and CD8^+^ lymphocytes [171]. Moreover, both lactate and H^+^ ions limit the function of macrophages by suppressing inflammasome formation [172] and promoting macrophage polarization [173]. It was reported that prolonged lactic acidosis leads to monocyte differentiation into pro-inflammatory macrophages of pro-tumour phenotype [174]. A detailed description of the interplay between lactate and immunity was recently reviewed by Caslin et al. [171].

## Evolution, fitness, and heterogeneity

**Fig. 5:**
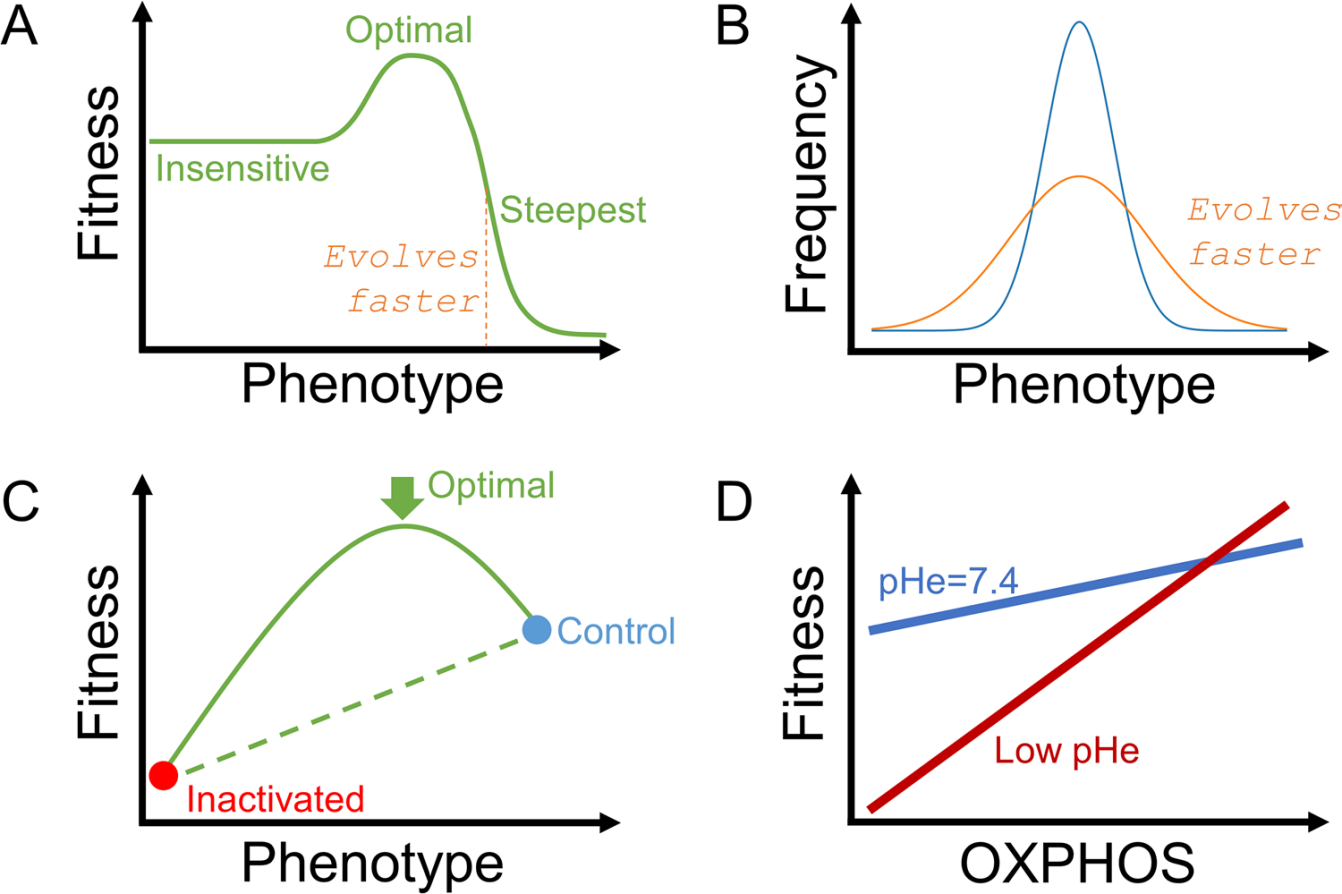
Understanding somatic evolution in terms of fitness and phenotypic heterogeneity. **A** Example of a fitness-phenotype relationship, showing a highly nonlinear behaviour. Optimal fitness is associated with an intermediate phenotype. The fastest rate of evolution is predicted around phenotypes that produce the steepest change in fitness. **B** Wider phenotypic variation is associated with higher rates of evolution. **C** Experiments that compare a control phenotype with a pharmacologically or genetically inactivated phenotype may miss important information about fitness. In this example, optimal fitness was attained at an intermediate phenotype and would not be detected with the canonical experimental approach. **D** The relationship between a phenotype and fitness can change in response to constraints placed by the environment. In this example, low pHe inhibits glycolysis, which forces the cell to rely more on oxidative phosphorylation (OXPHOS); this manifests as a steeper fitness-OXPHOS curve and therefore a higher rate of evolution

The discussion thus far has been limited to a notional population of ‘cancer’ and ‘normal’ cells forming a binary society in a tissue. However, an inalienable feature of cancer is its ability to evolve and pass on favourable characteristics to daughter cells, without being subject to the checks and controls that constrain normal cells. This process resembles the evolution of species in ecosystems, and its applicability to cancer was recognised by Peter Nowell in the 1970s [175]. The mathematical framework used to understand evolution is called game theory because a cell (player) takes on a strategy (phenotype) tailored to the strategies of other cells (co-players), with the aim to increase occupancy in the host tissues (assets) [176]. Evolution can be quantified in terms of phenotypic change over time [177]. This process can be described mathematically as the product of two components:

- The slope of the fitness/phenotype curve. This describes how the cell’s ability to survive a particular scenario is altered by a change in its phenotype. Changes to a phenotype that do not affect cell fitness will not drive evolution. In contrast, fitness that is conferred only by a narrow range of phenotypes will drive rapid evolution (Fig. 5A).
- The degree of variation in phenotype within the population of cells. Populations that show no heterogeneity will evolve slowly, whereas those with substantial diversity provide the substrate for evolution (Fig. 5B).

A faster pace of evolution is expected for populations that manifest broader phenotypic variation and for which even a small change in phenotype can meaningfully impact fitness to survive. Cancers that manifest these properties are more challenging to control therapeutically because a constant drug regime will eventually enrich the tumour in resistant cells. This situation is likely in tumours of high histological grading, wherein undifferentiated cells with stem-like properties have unlimited options to differentiate in response to selection pressures [178–181]. Despite the elegant simplicity of the equation governing the rate of phenotypic change, concepts such as ‘fitness sensitivity’ or ‘phenotypic heterogeneity’ are difficult to articulate in biological terms and interrogate experimentally. In the specific context of acidosis, many questions remain about the relationship between pHe, phenotype, fitness, and heterogeneity.

### ***Phenotype***

The emphasis on phenotype, rather than genotype, in somatic evolution is justified because selection favours particular functional outcomes, rather than their genetic instruction [177]. In the context of cancer cells, phenotype could refer to ensemble measures such as the proliferative rate or more elementary features such as glycolytic rate. For most normal cells in the body, it would be appropriate to quantify these phenotypes at pHe 7.4, i.e. the interstitial pHe of most tissues. The situation becomes more complicated in tumours, where pHe can be displaced from 7.4 and meaningfully influence phenotype. This raises the question of whether pHe sensitivity should be included in the definition of cancer phenotypes. Although pHe is ultimately influenced by the tissue’s genetic blueprint for metabolic acid production, extracellular buffering and volume, and blood perfusion, it is not normally considered an inheritable property in the same way that enzyme activity would be. Notwithstanding these questions, it is inevitable that mathematical representations of cancer will become more comprehensive and mechanistic and eventually include pHe sensitivity.

### ***Fitness***

The canonical approach to exploring the relationship between cellular fitness and phenotype has been pharmacological (e.g. inhibitors to block a specific process) or genetic (e.g. using overexpression or ablation to manipulate gene expression). In most studies, these investigations have been binary, e.g. comparing control with the inactivated state. While this approach has made way for some major discoveries, it actually contributes little to our understanding of somatic evolution. This is because the phenotype is a continuous variable, and its effect on cellular fitness can be highly nonlinear. As a result, probing fitness at only two phenotypic states sheds little light on fitness over the whole range of possible phenotypes. For example, the relationship between fitness and phenotype may produce optimal survival at an intermediate phenotype that would go undetected with the canonical pharmacological or genetic approach (Fig. 5C). Thus, measurements of fitness in the context of evolution must take into account that phenotype is a continuous variable and use appropriate methods to explore phenotypic responses with the necessary granularity. One method for smoothly grading phenotype exploits pHe sensitivity. Cancers may use pHe to fine-tune phenotype to a level that maximises the rate of evolution. pHe may also change the shape of the fitness-phenotype relationship by imposing constraints on cells. For example, low pHe inhibits glycolysis, which forces cells to rely more on oxidative phosphorylation. In such circumstances, cellular fitness becomes steeply dependent on mitochondrial metabolism, as compared to the alternative scenario at physiological pHe (Fig. 5D).

### ***Heterogeneity***

Phenotypic heterogeneity is possibly the least understood aspect of cancer cells. While it is tempting to use genotypic heterogeneity as a surrogate for phenotypic heterogeneity, the nontrivial coupling between genes and function largely invalidates this shortcut [182]. The conventional experimental approach of measuring phenotype has been to report its mean and standard error. This reporting convention focuses on conveying the average state of the population but fails to describe variation. Single-cell technologies, which have revolutionised transcriptomics [183–186], must now be extended for phenotyping. In reality, few phenotypes can be recorded at the necessary single-cell resolution. Moreover, a compounding issue is how to deconvolute experimental noise from genuine biological variance [187, 188]. Certain methods that have had a role in physiology research are compatible with single-cell measurements (e.g. cell fluorescence-based assays [189–191]) and an ambitious upscaling of these techniques can provide the information required to describe heterogeneity at the population level.

## Summary

The effects of acidity on cancer cells have often been described in terms of ill-defined cytotoxicity, akin to the actions of a ‘dirty’ yet potent drug. However, the role of acidity in tumours is much more nuanced than that of a cytotoxic drug. Firstly, acidity is an intrinsic feature of the tumour that is present throughout much of the process of carcinogenesis, albeit at varying degrees of intensity. Thus, acidity can exercise a persistent effect on cancer cells, which could be exploited therapeutically to produce a sustained shift in the tumour’s phenotypic landscape away from the most invasive type. This is in contrast to cytotoxic drugs that may exert a selection pressure during treatment episodes, but this effect cannot be sustained in the long term and may result in cancer recurrence, as predicted by mathematical models. Secondly, pH can influence the interaction between cancer cells and their host tissue, which has been highlighted by mathematical models as a viable treatment strategy. For example, low pHe targets host defences by suppressing immune surveillance. Releasing this inhibitory effect could empower the host tissue to eliminate cancer cells. Thirdly, pH is a fundamental property of the chemical microenvironment that is directly influenced by cells and feeds back on their survival prospects. Under acid stress, the selection of a fitter cancer subpopulation or adaptation towards a more compatible phenotype can endow cancer cells with a competitive advantage over host cells. Attenuating these pHe-driven processes could steer the cancer/host system away from the invasive trajectory. In this review, we have highlighted areas related to the acidosis that require further research attention, namely the heterogeneity of pH-related phenotypes, the aspects of fitness that are most sensitive to pH, and how best to achieve control over pHe *in vivo*. We have also explained the importance of adequate reporting standards and how to translate mathematical concepts borne from models into biological terms that could be tested experimentally.
